# Rare-variant collapsing analyses of arterial hypertension in the UK biobank

**DOI:** 10.1038/s41371-023-00829-7

**Published:** 2023-04-14

**Authors:** Bengt Zöller, Eric Manderstedt, Christina Lind-Halldén, Christer Halldén

**Affiliations:** 1 Center for Primary Health Care Research, Department of Clinical Sciences, Lund University and Region Skåne, Malmö, Sweden; 2 Department of Environmental Science and Bioscience, Kristianstad University, Kristianstad, Sweden

**Keywords:** Hypertension, Risk factors

## To the Editor:

*The importance of rare variation in hypertension is unclear. The present study uses UK biobank to analyse the association of rare coding variants to hypertension using gene collapsing data* (https://azphewas.com/ and https://app.genebass.org/). Arterial hypertension is an important contributor to the global burden of disease [1]. It is a multifactorial disease, with environmental, genetical, and social determinants [1]. Genetic loci associated with blood pressure comprises >30 genes, with rare variants resulting in monogenic forms of hypertension or hypotension [2]. Totally more than 1477 common single-nucleotide polymorphisms (SNPs) are associated with the blood pressure phenotype [2]. Still, SNPs identified in genome wide association studies (GWAS) of hypertension only explains around 27% of the 30–50% estimated heritability of blood pressure [2]. The availability of whole-genome sequencing (WES) data in large studies have enabled investigation of the contribution of rare variants to blood pressure and hypertension [3]. We have used two published UK biobank portals (https://azphewas.com/ and https://app.genebass.org/) [4, 5] to access gene collapsing analysis of rare variation for hypertension (Table 1). We did not obtain ethical approval because we used two public UK biobank portals (https://azphewas.com/ and https://app.genebass.org/) [4, 5]. The study by Wang et al. reported the relationships between rare protein-coding variants and 17,361 binary phenotypes using WES data from 269,171 UK Biobank exomes (https://azphewas.com/) [4]. Wang et al. used 12 different models to test the association between a total of 18,762 genes and 18,780 phenotypes [4]. These models included one recessive and ten dominant models. One synonymous variant model served as an empirical negative control [4]. A recent study by Karczewski et al. determined gene-based association in 394,841 UK biobanks exomes (https://app.genebass.org/) [5]. In total, Karczewski et al. performed 75,767 group tests for each of 4529 phenotypes, which include gene-based burden (mean), SKAT (variance), and SKAT-O (hybrid variance/mean) tests for predicted loss of function (pLOF) variants, missense (including low-confidence pLoF variants and in-frame insertions or deletions [indels]), synonymous variants, and the combination pLoF or missense group (not displayed in https://app.genebass.org) [5]. Detailed descriptions of the databases are found in respective publication and websites (https://azphewas.com/ and https://app.genebass.org/) [4, 5]. The significance levels used in the two portals and published studies were stringent [4, 5]. In order not to discard potential candidate genes, we present genes with *p* < 0.05/20,000 genes = 2.5 × 10^–6^ commonly used for WES studies. In Table 1 only the genes with genome-wide significant results are shown with p-values for the most significant model. Rare variation was linked to essential hypertension in seven previously GWAS linked hypertension genes (*CACNA1D*, *NR3C2*, *NOS3*, *DNMT3A*, *ENPEP*, *GUCY1A1*, and *UMOD*) (https://www.ebi.ac.uk/gwas/). Thus, not only common variation but also rare variation in these genes is important for essential hypertension. The same is true for three genes linked to blood pressure levels, but not hypertension, in GWAS studies (*COL4A4*, *DBH*, and *PKD1*) (https://www.ebi.ac.uk/gwas/). However, the *DBH* gene has also been linked to orthostatic hypotension 1, due to DBH deficiency and the *COL4A4* and *PKD1* genes have also been associated with kidney disease (Alport syndrome, familial benign hematuria and polycystic kidney disease 1) (https://www.omim.org/). The *ASXL1* (ASXL Transcriptional Regulator 1) gene is associated with myelodysplastic syndrome (MDS), Bohring-Opitz syndrome, and systemic mastocytosis, which may cause hypotension (https://www.omim.org and https://varsome.com). Clonal haematopoiesis of indeterminate potential (CHIP), linked to *DNMT3A*, *TET2*, and *ASXL1* mutations, is a recently described phenomenon preceding MDS that has been associated with cardiovascular diseases [6, 7]. However, it is unclear why *ASXL1* is associated with essential hypertension and CHIP *DNMT3A* mutations have been associated with hypertension in human patients and mice [8]. Both the *PKD1* and *UMOD* (uromodulin) were linked to hypertensive kidney disease. *UMOD* is known to be associated with autosomal dominant tubulointerstitial kidney disease (https://www.omim.org/). No gene was significant for hypertensive heart disease.

**Table 1:** Results of gene collapsing analysis of rare variants for arterial hypertension according to three-digit ICD-10 codes.

	Genebass (LOF)	Genebass (missense/LC)	Astra Zeneca portal
(International Classification of Diseases 10th Revision)	*p* < 2.5 × 10^–6^ (number of variants [N] and cumulative allele frequency [CAF])	*p* < 2.5 × 10^–6^ (number of variants [N] and cumulative allele frequency [CAF])	*p* < 2.5 × 10^–6^ (cumulative allele frequency among cases [CAFac])
I10 Essential (primary) hypertension (Genebass *N* cases = 98,167 and *N* controls = 296,674) (Astra Zeneca *N* cases = 98,167 and *N* controls = 296,674)	*PKD2**p* = 2.76e-8 (*N* = 39, CAF = 1.49e-4) COL4A4*p* = 2.23e-7 (*N* = 83, CAF = 7.94e-4)	*CACNA1D*p* = 5.66e-8 (*N* = 844, CAF = 0.0288) *DBH**p* = 1.15e-6 (*N* = 392, CAF = 0.259) *NR3C2*p* = 1.76e-6 (*N* = 406, CAF = 0.895)	*PKD1**p* = 5.23e-13 (CAFac = 0.0006) *ASXL1**p* = 6.72e-11 (CAFac = 0.0015) *NOS3*p* = 1.19e-9 (CAFac = 0.005) *DNMT3A*p* = 6.52e-9 (CAFac = 0.0038) *PKD2**p* = 2.90e-8 (CAFac = 0.0003) *ENPEP*p* = 6.65e-8 (CAFac = 0.0274) *GUCY1A1*p* = 2.46e-7 (CAFac = 0.0007)
I11 Hypertensive heart disease	NS	NS	NS
I12 Hypertensive renal disease	*UMOD*p* = 5.8e-10 (*N* = 51, CAF = 5.17e-4)	NS	*PKD1**p* = 3.50e-40 (CAFac = 0.0171) *UMOD*p* = 2.02e-6 (CAFac = 0.003)
I13 Hypertensive heart and renal disease	ND	ND	NS
I15 Secondary hypertension	*SSTR2**p* = 1.02e-7 (*N* = 19, CAF = 4.19e-5)	*ATOH1**p* = 5.08e-7 (*N* = 213, CAF = 0.0136)	*PKD1**p* = 8.16e-13 (CAFac = 0.0327)

For Genebass portal (https://app.genebass.org), the highest values for SKAT-O, SKAT, or burden tests are shown. For Astra Zeneca portal (https://azphewas.com) the highest *p*-value of the 12 tested models is shown.

LoF: High-confidence Loss of function variants indicated by LOFTEE [5]. Missense/LC: Missense variants are grouped with in-frame insertions and deletions, as well as low-confidence LoF variants filtered out by LOFTEE [5]. The latter have a frequency spectrum consistent with missense variation and affects a set of amino acids in a similar way [5].

*NS* no genome-wide significant gene, *ND* not determined.

*GWAS associated hypertension genes (https://www.ebi.ac.uk/gwas/). COL4A4 is associated with Alport syndrome and Hematuria, familial benign (https://www.omim.org/) but also in GWAS with systolic blood pressure, diastolic blood pressure (https://www.ebi.ac.uk/gwas/). *DBH* is associated with orthostatic hypotension 1, due to DBH deficiency (=https://www.omim.org/) but also in GWAS with systolic blood pressure, diastolic blood pressure, mean arterial pressure (https://www.ebi.ac.uk/gwas/). *PKD1* is associated with polycystic kidney disease 1 (https://www.omim.org/) but also in GWAS with systolic blood pressure and pulse pressure (https://www.ebi.ac.uk/gwas/). PKD2 is associated with polycystic kidney disease 2 (https://www.omim.org/). *ASXL1* is associated with myelodysplastic syndrome, systemic mastocytosis, and Bohring-Opitz syndrome (https://www.omim.org/ and https://varsome.com).

Union was used to define phenotypes for https://azphewas.com. Only genome wide significant associated genes are shown (best model) i.e., <0.05/20,000 genes = 2.5 × 10^–6^ commonly used for whole exome sequencing (WES) studies.

Rare variation of the *SSTR2*, *ATOH1*, and *PKD1* genes were associated with secondary hypertension (Table 1). Thus, the *PKD1* gene was linked to both essential hypertension and secondary hypertension. The *SSTR2* gene (somatostatin receptor 2) has previously been suggested to be a candidate gene for hypertension but has not formally been linked to hypertension. Thus, the *SSTR2*, *ATOH1* and *ASXL1* genes are all novel hypertension candidate genes. No human cardiovascular disease has been linked to the *ATOH1* (Atonal BHLH Transcription Factor 1) gene (https://www.omim.org/) and it is unclear how this gene could be linked to secondary hypertension. However, the involvement of the cerebellum in cardiopulmonary function such as blood pressure regulation has long been established and the *ATOH1* gene is important for cerebellar development in mice [9, 10]. *SSTR2* has been linked to acromegaly (https://www.genecards.org/) and hypertension is a complication to acromegaly (https://my.clevelandclinic.org/).

The gene collapsing analysis in UK biobank study by Wang et al. [4] and Karczewski et al. [5] show that rare variation in 15 genes contribute to primary or secondary hypertension. Interestingly, genes with rare variation contributing to hypertension in many cases also have common variants contributing to hypertension or blood pressure, suggesting that targeted resequencing of GWAS associated genes in hypertensive patients might be valuable. The finding that rare variation in four genes (*COL4A4*, *PKD1*, *PKD2*, and *UMOD*) previously has been linked to kidney disease (https://www.genecards.org/) also emphasize the importance of the kidneys in the pathogenesis of essential hypertension. A strength of UK biobank is the very large study size, though it is possible that even larger study sizes may reveal additional clinically relevant associations.

The finding that the gene collapsing analyses in essential hypertension identified variation in genes (*PKD1*, *PKD2*, *COL4A4*, *UMOD*, *CACNA1D*, and *NR3C2*) that are possible causes of undiagnosed secondary hypertension is worthy a special comment. Essential, primary, or idiopathic hypertension is defined as high blood pressure when secondary causes such as renovascular disease, renal failure, pheochromocytoma, aldosteronism, or mendelian forms (monogenic) are not present [11]. The present findings in UK biobank may suggest that genetic analysis may lead to better identification of patients with secondary hypertension [4, 5]. For instance, mutations in the *CACNA1D* (primary aldosteronism) and *NR3C2* (Geller syndrome) genes are known causes of monogenic hypertension [12]. All the variants included in the gene collapsing pLOF analyses of the *PKD1* and *COL4A4* genes are most likely pathogenic. Some have previously been associated with disease; for instance, a deletion in the *PKD1* (p.Asn720IlefsTer17, *rs757757289*) has been reported to be associated with polycystic kidney disease. A detailed description of the included variants in each gene collapsing model are shown in both portals (https://azphewas.com/ and https://app.genebass.org/).

In Table 1, the number of variants included in gene collapsing analysis and the cumulative allele frequency in the population is shown for Genebass while the cumulative allele frequency among cases in the Astra Zeneca portal is shown (https://app.genebass.org/ and https://azphewas.com/) [4, 5]. For most genes, the number of individuals with a qualifying variant is limited, though gene collapsing analysis in some instances adds up to a large number that might make an important contribution to heritability. However, we have not been able to determine the contribution to heritability.

In conclusion, rare variation in 15 genes were associated with hypertension in UK biobank. This suggests that rare variation contributes not only to secondary but also essential hypertension. The association with *SSTR2*, *ASXL1*, and *ATOH1* may suggest potential novel mechanism in the pathogenesis of hypertension that needs to be to further elucidated.

## Data Availability

Data are publicly available (https://azphewas.com/ and https://app.genebass.org/) [4, 5].
